# Supplementary material for: Mapping the evolution of fertility support policies in China: A content and instrumental analysis
Source: PLoS One. 2025 Oct 9;20(10):e0332137. doi: 10.1371/journal.pone.0332137 (PMC12510515; doi:10.1371/journal.pone.0332137)
Supplement: S1 Appendix — (ZIP) [file pone.0332137.s001.zip › S1 Appendix. 226 original policy documents/59-国务院办公厅关于印发国家贫困地区儿童发展规划(2014—2020年)的通知(FBM-CLI-2-241286).doc]

国务院办公厅关于印发国家贫困地区儿童发展规划(2014—2020年)的通知

**发布部门：** [国务院办公厅](https://www.pkulaw.com/law/chl?Aggs.IssueDepartment=601) [机构沿革](javascript:void(0))

**发文字号：**国办发〔2014〕67号

**发布日期：**2014.12.25

**实施日期：**2014.12.25

**时效性：** [现行有效](https://www.pkulaw.com/law/chl?Aggs.TimelinessDic=01)

**效力级别：** [国务院规范性文件](https://www.pkulaw.com/law/chl?Aggs.EffectivenessDic=XC0203)

**法规类别：** [贫困地区经济开发](https://www.pkulaw.com/law/chl?Aggs.Category=02307)

国务院办公厅关于印发国家贫困地区儿童发展规划（2014-2020年）的通知
（国办发〔2014〕67号）

各省、自治区、直辖市人民政府，国务院各部委、各直属机构：
　　《国家贫困地区儿童发展规划（2014-2020年）》已经国务院同意，现印发给你们，请结合实际认真贯彻执行。

国务院办公厅
2014年12月25日

国家贫困地区儿童发展规划
（2014-2020年）

　　儿童发展关系国家未来和民族希望，关系社会公平公正，关系亿万家庭的幸福。改革开放特别是进入21世纪以来，我国儿童健康、教育水平明显提高，儿童生存、发展和受保护的权利得到有力保障，提前实现了联合国千年发展目标。但总体上看，我国儿童事业发展还不平衡，特别是集中连片特殊困难地区的4000万儿童，在健康和教育等方面的发展水平明显低于全国平均水平。进一步采取措施，促进贫困地区儿童发展是切断贫困代际传递的根本途径，是全面建成小康社会的客观要求，也是政府提供基本公共服务的重要内容。为进一步促进贫困地区儿童发展，编制本规划。
　　**一、**总体要求
　　（一）指导思想。以邓小平理论、“三个代表”重要思想、科学发展观为指导，深入贯彻党的十八大和十八届二中、三中、四中全会精神，认真落实党中央、国务院决策部署，坚持儿童优先原则，坚持儿童成长早期干预基本方针，以健康和教育为战略重点，以困难家庭为主要扶持对象，加大统筹协调、资源整合和推进发展力度，实行政府直接提供服务和向社会力量购买服务相结合的工作机制，切实保障贫困地区儿童生存和发展权益，实现政府、家庭和社会对贫困地区儿童健康成长的全程关怀和全面保障。
　　（二）实施范围。集中连片特殊困难地区680个县从出生到义务教育阶段结束的农村儿童。
　　（三）总体目标。到2020年，集中连片特殊困难地区儿童发展整体水平基本达到或接近全国平均水平。
　　--保障母婴安全。孕产妇死亡率下降到30/10万，婴儿和5岁以下儿童死亡率分别下降到12‰和15‰。出生人口素质显著提高。
　　--保障儿童健康。5岁以下儿童生长迟缓率降低到10%以下，低体重率降低到5%以下，贫血患病率降低到12%以下。以乡镇为单位适龄儿童国家免疫规划疫苗接种率达到并保持在90%以上。中小学生体质基本达到《国家学生体质健康标准》。特殊困难儿童的福利、关爱体系更加健全。
　　--保障儿童教育。学前三年毛入园率达到75%。义务教育巩固率达到93%，教育总体质量、均衡发展水平显著提高。视力、听力、智力残疾儿童少年义务教育入学率达到90%。

　　**二、**主要任务
　　（一）新生儿出生健康。
　　1.加强出生缺陷综合防治。落实出生缺陷综合防治措施，实施国家免费孕前优生健康检查项目，推进增补叶酸预防神经管缺陷等项目，做好孕期产期保健，逐步开展相关的免费筛查、诊断试点项目，提高出生人口素质。开展新生儿先天性甲状腺功能减低症、苯丙酮尿症、听力障碍等疾病筛查服务，加强儿童残疾筛查与康复的衔接，提高筛查确诊病例救治康复水平。
　　2.加强孕产妇营养补充。开展孕前、孕产期和哺乳期妇女营养指导，制定孕产期妇女营养素补充标准，预防和治疗孕产妇贫血等疾病，减少低出生体重儿。
　　3.加强孕产妇和新生儿健康管理。加强高危孕妇的识别与管理、早产儿的预防与干预，提高孕产妇和儿童系统管理率。继续实施农村孕产妇住院分娩补助项目，做好与新型农村合作医疗和医疗救助制度的有效衔接，加大贫困地区孕产妇住院分娩保障力度。建立危重孕产妇和新生儿急救绿色通道及网络。
　　4.加强优生优育宣传教育。通过广播电视、公益广告、集中教育等多种方式，深入开展“婚育新风进万家活动”、“关爱女孩行动”、“新农村新家庭计划”等宣传活动。结合基本公共卫生和计划生育服务，针对贫困地区儿童发展特点，设计开发优生优育等方面的出版物和宣传品。教育、卫生计生部门要共同组织开展学生青春期教育。残联、卫生计生部门要共同组织开展残疾预防宣传活动。通过现场专题讲座、远程教育和多媒体专题辅导等方式，向育龄群众和孕产妇传授优生优育专业知识。
　　（二）儿童营养改善。
　　1.改善婴幼儿营养状况。倡导0-6个月婴儿纯母乳喂养，加强母乳喂养宣传及相关知识培训。扩大贫困地区困难家庭婴幼儿营养改善试点范围，以低保家庭、低保边缘家庭为重点，逐步覆盖到集中连片特殊困难地区的680个县，预防儿童营养不良和贫血。
　　2.完善农村义务教育学生营养改善工作机制。各地要进一步落实农村义务教育学生营养改善计划管理责任和配套政策，切实加强资金使用和食品安全管理。因地制宜新建或改扩建农村义务教育学校伙房或食堂等设施，逐步以学校供餐替代校外供餐。继续支持各地开展义务教育阶段学生营养改善试点。有条件的地方可结合实际，以多种方式做好学前教育阶段儿童营养改善工作。
　　3.提高儿童营养改善保障能力。建立儿童营养健康状况监测评估制度。加强对各级妇幼保健机构、计划生育服务机构、疾病预防控制机构和基层医疗卫生机构人员的营养改善技能培训，提高预防儿童营养性疾病指导能力。加强对中小学幼儿园教师、食堂从业人员及学生家长的营养知识宣传教育，引导学生及其家庭形成健康饮食习惯。鼓励社会团体和公益组织积极参与儿童营养改善行动。
　　（三）儿童医疗卫生保健。
　　1.完善儿童健康检查制度。对儿童生理状况、营养状况和常见病进行常规检查，建立儿童体检档案，定期对身高、体重、贫血状况等进行监测分析。将入学前儿童健康体检纳入基本公共卫生服务，由基层医疗卫生机构免费提供；义务教育阶段学生按中小学生健康检查基本标准进行体检，所需费用纳入学校公用经费开支范围。
　　2.加强儿童疾病预防控制。切实落实国家免疫规划，为适龄儿童免费提供国家免疫规划疫苗接种服务，开展针对重点地区重点人群脊髓灰质炎、麻疹等国家免疫规划疫苗补充免疫或查漏补种工作。落实碘缺乏病、地方性氟中毒、大骨节病防治措施，有效控制地方病对儿童健康的危害。各级妇幼保健机构要加强新生儿健康和儿童疾病预防服务，加强儿童视力、听力和口腔保健工作，预防和控制视力不良、听力损失、龋齿等疾病发生。
　　3.提高儿童基本医疗保障水平。完善城乡居民基本医疗保险制度，通过全民参保登记等措施，使制度覆盖全体儿童。全面推进城乡居民大病保险，逐步提高儿童大病保障水平。完善城乡医疗救助制度，加大儿童医疗救助力度，做好与大病保险制度、疾病应急救助制度的衔接，进一步提高儿童先天性心脏病、白血病、唇腭裂、尿道下裂、苯丙酮尿症、血友病等重大疾病救治费用保障水平。
　　4.加强儿童医疗卫生服务能力建设。加强妇幼保健机构、妇产医院、儿童医院、综合性医院妇产科儿科和计划生育服务体系建设，提高基层医疗卫生机构孕产期保健、儿童保健、儿童常见病诊治、现场急救、危急重症患儿救治和转诊能力。加强助产技术、儿童疾病综合管理、新生儿复苏等适宜技术培训和儿童临床疾病诊治及护理培训，提高妇幼保健人员、计划生育技术人员和医护人员服务能力和水平。寄宿制学校或者600人以上的非寄宿制学校要设立卫生室（保健室），配备人员器材。县级政府要建立健全学校突发公共卫生事件应急管理机制。
　　5.保障学生饮水安全和学校环境卫生。结合实施国家农村饮水安全工程，多渠道加大投入，统筹考虑和优先解决集中连片特殊困难地区农村学校饮水问题，实现供水入校。对无法接入公共供水管网的学校，就近寻找安全水源或实行自备水井供水。定期检测学校饮用水，保障水质达标。加强农村学校卫生厕所、浴室等生活设施建设，为学生提供健康生活环境，从小培养文明生活习惯。
　　6.加强体育和心理健康教育。加强学校体育设施建设和体育器材配备，在基层公共体育设施建设中统筹规划学校体育设施。切实加强学校体育工作，严格落实每天锻炼一小时的要求，大力开展符合农村特点的体育活动和群众性体育项目竞赛。建立健全儿童心理健康教育制度，重点加强对留守儿童和孤儿、残疾儿童、自闭症儿童的心理辅导。加强班主任和专业教师心理健康教育能力建设，使每一所学校都有专职或兼职的心理健康教育教师。在农村义务教育学校教师特设岗位计划和中小学教师国家级培训计划中加大对体育和心理健康教育骨干教师的补充和培训力度。
　　（四）儿童教育保障。
　　1.开展婴幼儿早期保教。依托幼儿园和支教点，为3岁以下儿童及其家庭提供早期保育和教育指导服务。采取多种形式宣传普及早期保教知识，鼓励媒体开办公益性早教节目（栏目）。建立城乡幼儿园对口帮扶机制，组织专家和有经验的志愿者到边远地区开展科学早教服务。
　　2.推进学前教育。坚持政府主导、社会参与、公办民办并举，多种形式扩大贫困地区普惠性学前教育资源。加大中央财政学前教育发展重大项目、农村学前教育推进工程和省级学前教育项目对集中连片特殊困难地区的倾斜支持力度。扩大实施中西部农村偏远地区学前教育巡回支教试点，在人口分散的山区、牧区设立支教点，通过政府购买服务和动员社会力量招募大中专毕业生志愿者开展巡回支教，中央财政予以适当补助。在需要的民族地区加强学前双语教育。地方政府要依法落实相关政策，稳定贫困地区幼儿园教职工队伍。完善学前教育资助制度，帮助家庭经济困难儿童、孤儿和残疾儿童接受普惠性学前教育。
　　3.办好农村义务教育。明确各地巩固义务教育目标，将义务教育控辍保学责任分解落实到地方各级政府、有关部门和学校，并作为教育督导重点内容。推动各地制定义务教育阶段学校标准化的时间表、路线图，解决农村义务教育中寄宿条件不足、大班额、上下学交通困难、基本教学仪器和图书不达标等突出问题。支持各地制定实施贫困地区教师队伍建设规划，统筹教师聘任（聘用）制度改革、农村义务教育学校教师特设岗位计划、中小学教师国家级培训计划、教师合理流动、对口支援等政策，系统解决贫困地区合格教师缺乏问题。对已实施集中连片特殊困难地区乡、村学校和教学点教师生活补助政策的地方，中央财政予以奖补。综合考虑提高教育质量、物价上涨、信息化教育和学生体检等需要，适时提高农村义务教育学校生均公用经费标准。
　　4.推进农村学校信息化建设。大力推进宽带网络校校通、优质资源班班通、网络学习空间人人通。各地要结合实施“宽带中国”战略和贫困村信息化工作，积极推动为贫困地区中小学接入宽带网络。将校内信息基础设施建设列入学校新建、改扩建和薄弱学校改造等项目建设内容。加强教师信息技术应用能力培训，建立面向农村的数字教育资源应用平台，扩大优质数字教育资源共享范围，提升农村学校教学质量。
　　5.保障学生安全成长。学校要建立面向全体学生和家长的安全教育制度、安全管理制度和应急信息通报报告制度，落实校园安全责任制。改善学校安全条件，建设符合安全标准的校舍、围墙、栅栏等设施，加强视频监控、报警设施和安全防护设备的配备，落实专门人员做好相关工作。寄宿制学校要完善教师值班制度，配备必要的生活管理教师，落实学生宿舍安全管理责任。预防和控制儿童意外伤害。对儿童人身伤害案件依法从重查处。采取就近入学、建设寄宿制学校、发展公共交通、提供校车服务等措施，方便学生安全上下学。净化校园及周边治安环境，维护学生安全和校园稳定。
　　（五）特殊困难儿童教育和关爱。
　　1.完善特殊困难儿童福利制度。重点支持在人口较多和孤儿数量多的县（市）建设一批儿童福利院或社会福利机构儿童部。探索适合孤儿身心发育的养育模式，鼓励家庭收养、寄养和社会助养。落实好为孤儿、艾滋病病毒感染儿童发放基本生活费的政策，探索建立其他困境儿童基本生活保障制度。为0-6岁残疾儿童提供康复补贴。保证适龄孤儿进入相应的学校就读，将义务教育阶段的孤儿寄宿生全面纳入生活补助范围。推进残疾人康复和托养设施建设，基本实现每个地级城市都建有一所专业化残疾人康复机构，并配备儿童听力语言康复、智力康复、孤独症康复、脑瘫康复等设施。
　　2.保证残疾儿童受教育权利。逐步提高特殊教育学校生均公用经费标准，对残疾学生实行免学杂费、免费提供教科书、补助家庭经济困难寄宿生生活费等政策，进一步加大残疾学生资助力度。按实际需求配足配齐特殊教育教师，落实特殊教育教师倾斜政策，逐步提高工资待遇水平。加强特殊教育教师培养培训，提高专业化水平。积极创造条件，扩大普通学校随班就读规模，鼓励农村残疾儿童就近接受教育。积极推进全纳教育，使每个残疾儿童都能接受合适的教育。学校和医疗机构要相互配合推进医教结合，实施有针对性的教育、康复和保健。建立和完善服务机制，统筹学校、社区和家庭资源，在有条件的地区为不能进校就读的重度残疾儿童少年提供送教上门服务。支持和指导儿童福利机构特教班建设，落实儿童福利机构特殊教育教师的相应待遇。
　　3.完善儿童社会保护服务体系。充分发挥现有流浪儿童救助保护制度的作用，探索建立儿童社会保护“监测预防、发现报告、帮扶干预”反应机制，推动建立以家庭监护为基础、社会监督为保障、国家监护为补充的监护制度。将儿童保护纳入社区管理和服务职能，动员社区学校、幼儿园、医院及其他社会组织参与儿童保护工作。各地可结合实际，依托城乡社区现有公共服务设施建立儿童活动场所。建立儿童社会保护工作机制和服务网络，将救助保护机构扩展为社会保护转介平台，面向社会开展儿童权益保护服务，最大限度改善困境儿童生存状况。进一步加大劳动保障监察执法力度，努力消除使用童工等违法行为。
　　4.健全留守儿童关爱服务体系。加强农村寄宿制学校建设，优先满足留守儿童就学、生活和安全需要。学校对留守儿童受教育实施全程管理，注重留守儿童心理健康教育和亲情关爱，及早发现和纠正个别留守儿童的不良行为。强化父母和其他监护人的监护责任并提高其监护能力，加强家庭教育指导服务，引导外出务工家长以各种方式关心留守儿童。依托现有机构和设施，健全留守儿童关爱服务体系，组织乡村干部和农村党员对留守儿童进行结对关爱服务。开展城乡少年手拉手等活动，支持为农村学校捐建手拉手红领巾书屋，建设流动少年宫，丰富留守儿童精神文化生活。

　　**三、**保障措施
　　（一）加强整体规划和资源整合。在与现有规划、政策、项目等充分衔接基础上，按照整合资源、集中财力、聚焦重点的原则，统筹规划贫困地区儿童发展政策，充分利用一般性转移支付、现有项目资金、对口支援项目等，按照规划目标集中调配资源，支持贫困地区儿童发展。各有关部门要按照统一部署，把规划主要任务和重点工程纳入本部门发展规划、年度计划，并给予优先安排。
　　（二）落实经费投入和管理。建立健全以财政投入为主、社会力量参与、家庭合理分担的贫困地区儿童发展经费投入机制。中央和地方财政进一步加大儿童发展投入力度。对支持儿童发展的社会公益项目，有关部门和地方政府要加强协调支持，依法落实税收优惠政策。建立健全管理制度，确保用于贫困地区儿童发展的各项资金使用安全、规范和有效。审计部门要加强对贫困地区儿童发展专项资金和政府购买服务经费的审计。财政部门要加强经费监管和绩效考评。加大资金管理使用公开力度，接受社会监督。对管理中出现的问题，要依法追究相关责任人的责任。
　　（三）创新公共服务提供方式。鼓励采取政府向社会力量购买服务的方式实施儿童发展项目，对适合市场化方式提供的事项，交由具备条件、信誉良好的群团组织、社会组织和企业等承担，并和社会公益项目有机结合，扩大公共服务供给。规范政府购买服务程序，按照公开、公正、公平的原则，以竞争择优的方式确定承接主体，并通过委托、承包、采购等方式购买儿童健康、教育、福利、安全等领域的公共服务。严格政府购买服务资金管理，在既有预算中统筹安排，以事定费，规范透明。
　　（四）发挥社会力量作用。积极引导各类公益组织、社会团体、企业和有关国际组织参与支持贫困地区儿童发展。鼓励志愿者到贫困地区开展支教、医疗服务和宣传教育工作。加强政府相关部门、学校、公共卫生和医疗机构与家庭、社区的沟通，鼓励家长参与儿童发展项目的实施。

　　**四、**组织实施
　　（一）落实地方政府责任。贫困地区儿童发展工作在国务院统一领导下，实行地方为主、分级负责、各部门协同推进的管理体制。省级政府负责统筹组织，制订实施工作方案和推进计划。地市级政府要加强协调指导，督促县级政府和有关部门明确责任分工，细化政策措施。县级政府要统筹整合各方面资源，落实各项具体政策和工作任务，创新管理和运行方式，切实提高支持政策和项目的执行效率。
　　（二）明确部门职责分工。发展改革部门要将贫困地区儿童发展纳入国民经济和社会发展总体规划，完善贫困地区妇幼保健和儿童医疗、教育、福利服务等基础设施建设。财政部门要统筹安排财政资金，加强经费监管。教育、卫生计生、民政、公安、工业和信息化、水利、扶贫、妇儿工委等部门要切实履行职责，并加强协调和指导。妇联、共青团、残联等单位要积极参与做好促进儿童发展各项工作。
　　（三）开展监测评估。各级政府对规划实施进展、质量和成效进行动态监测评估，将规划重点任务落实情况作为政府督查督办重要事项，并将结果作为下一级政府绩效考核重要内容。建立健全评估机制，开展第三方评估。充分发挥卫生计生、教育和社会政策等领域专家作用，开展贫困地区儿童发展重大问题决策咨询。
　　（四）营造良好氛围。广泛宣传促进贫困地区儿童发展的重要性和政策措施，做好政策解读、回应群众关切，宣传先进典型、推广经验做法，动员全社会关心支持贫困地区儿童发展，为规划实施创造良好社会环境。

© 北大法宝：（[www.pkulaw.com](https://www.pkulaw.com/)) 专业提供法律信息、法学知识和法律软件领域各类解决方案。北大法宝为您提供丰富的参考资料，正式引用法规条文时请与标准文本核对。
欢迎查看所有[产品和服务](https://www.pkulaw.com/helps/2.html)。法宝快讯：[如何快速找到您需要的检索结果？](https://www.pkulaw.com/helps/70.html)[法宝](https://www.pkulaw.com/helps/69.html)V6有何新特色？

扫描二维码阅读原文

**原文链接：**<https://www.pkulaw.com/chl/079bf6d08a1d48b4bdfb.html>
